# Dementia risk in patients with heart disease and depression

**DOI:** 10.1590/1980-5764-DN-2023-0024

**Published:** 2023-10-23

**Authors:** Daniel Denisenko, Gladys Ekong, Harlan Spotts

**Affiliations:** 1 Western New England University, College of Pharmacy and Health Sciences, Springfield, MA, USA.; 2 Western New England University, College of Business, Springfield, MA, USA.

**Keywords:** Aging, Dementia, Depression, Heart Diseases, Alzheimer Disease, Envelhecimento, Demência, Depressão, Cardiopatias, Doença de Alzheimer

## Abstract

The high prevalence of Alzheimer's disease and dementia is a growing concern for healthcare systems and patients.

**Objective:**

The primary objective of our study was to assess the association of depression and heart disease on the risk of dementias like Alzheimer's disease or vascular dementia in patients.

**Methods:**

This retrospective study used electronic health records data that was provided by the HealthVerity™ Marketplace. The characteristics of the patient population were recorded and the risk of dementia was examined using adjusted logistic regression models.

**Results:**

The analysis included 49,735 participants and revealed that patients who have heart disease or depression had a higher risk of dementia. Patients who had both heart disease and depression were over three times more likely to have dementia and Alzheimer's disease, and over five times more likely to have vascular dementia compared to patients who only have a diagnosis of heart disease. Depression was associated with a fourfold increase in the risk of dementia. Participants with a diagnosis of most types of heart disease as well as depression had increased risk for developing dementia.

**Conclusion:**

This study revealed that patients with both heart disease and depression had increased odds of having dementia as well as vascular dementia and Alzheimer's disease. These findings may serve to support policies and healthcare decision-making to increase preventive measures for dementia and Alzheimer's disease among patients with both depression and heart disease.

## RESUMO.

A alta prevalência da doença de Alzheimer e demência é uma preocupação crescente para os sistemas de saúde e pacientes.

**Objetivo:**

O objetivo primário do nosso estudo foi avaliar a associação entre depressão e doença cardíaca no risco de demências como a doença de Alzheimer ou demência vascular.

**Métodos:**

Este estudo retrospectivo utilizou dados de registros eletrônicos de saúde fornecidos pelo *HealthVerity*™ *Marketplace*. As características da população de pacientes foram registradas e o risco de demência foi examinado usando modelos de regressão logística ajustados.

**Resultados:**

A análise incluiu 49.735 participantes e revelou que pacientes com doenças cardíacas ou depressão apresentavam maior risco de demência. Os pacientes que tinham doença cardíaca e depressão tinham mais de três vezes mais chances de ter demência e doença de Alzheimer, e mais de cinco vezes mais chances de ter demência vascular em comparação com pacientes que tinham apenas um diagnóstico de doença cardíaca. A depressão foi associada a um aumento de quatro vezes no risco de demência. Os participantes com diagnóstico da maioria dos tipos de doenças cardíacas, bem como depressão, apresentaram risco aumentado de desenvolver demência.

**Conclusão:**

Este estudo revelou que pacientes com doenças cardíacas e depressão tinham maior probabilidade de ter demência, bem como demência vascular e doença de Alzheimer. Essas descobertas podem servir para apoiar as políticas e a tomada de decisões de saúde para aumentar as medidas preventivas para demência e doença de Alzheimer entre pacientes com depressão e doenças cardíacas.

## INTRODUCTION

The past few decades have seen a substantial and historic rise in life expectancy and a substantial decrease in mortality rates. However, despite this positive development, a high prevalence of dementias including Alzheimer's disease (AD) has been observed among geriatric patients. Dementia is a medical term used to refer to a series of conditions characterized by the impairment of thinking ability, memory, decision making, and performing activities of daily living^1^. Dementia is an irreversible and progressive condition with a higher prevalence among geriatric patients^1^. The most prevalent form of which is Alzheimer's disease; other forms include vascular dementia, Parkinson's dementia, Lewy body dementia, and frontotemporal dementia. The prevalence of dementia has increased as the life expectancy in the US has improved due to the availability of effective treatment options for chronic disease management. By 2050, it is projected that 152 million people will have dementia worldwide (up from a projected 50 million in 2020) and the current global cost of the condition now surpasses $1 trillion annually^2^. Dementia is associated with the presence of other health conditions; primarily heart disease and depression.

Heart disease, also commonly referred to as cardiovascular disease, is a variety of different ailments and medical conditions that affect the heart and the blood vessels. Varieties of heart disease include coronary artery disease (CAD), peripheral artery disease (PAD), stroke, myocardial infarction (MI), and heart failure (HF). Heart disease is a principal cause of death and disability in the United States as well as worldwide; deaths attributed to cardiovascular disease in the U.S. eclipsed 800,000 in the year 2016 and an estimated half of Americans have some form of the condition^3^. Studies have examined the impact of cardiovascular diseases on dementia and have observed an elevated incidence of this condition in patients who have cardiovascular disease^4–6^. A 2020 study observed a higher risk of developing dementia in patients who had heart failure; however Alzheimer's disease risk was unclear^7^.

Depression is another known risk factor for the development of dementia and has a high prevalence in patients who have been diagnosed with dementia or AD^8^. It is a psychological disorder principally characterized by an abnormally and persistently diminished mood and feeling of well-being and has numerous variations and severities. Studies have shown that depression developing early in life has a two-fold or greater risk of dementia development later in life^9^. Positive associations have also been formed between the emergence of depression later in life and the risk of dementia^10^. However, the results are more conflicting and the nature of the effect is still unclear. Recent research studies have found that many cases of dementia may, in fact, possibly be preventable or delayed through the treatment of depression^11^. While more research is needed, the potential to delay or even prevent dementia through antidepressant therapy would lead to a major shift in public health policy towards depression and could substantially alleviate the societal and economic burden of dementia.

Heart disease is the most common illness worldwide with an age-adjusted prevalence rate of 7,354.1 per 100,000 in 2020, contributing to the bulk of human mortalities every year. The reported rates of depression are also rapidly on the rise and have been so for the past few decades^12^. Thus, it is imperative to conduct more research into the risk these conditions pose on the development of various forms of dementia like Alzheimer's or vascular dementia, as this is vital for future health research and health policy decision-making. The primary objective of our study was to assess the association of depression and heart disease on the risk of dementias like Alzheimer's disease or vascular dementia in patients.

## METHODS

### Sample

This retrospective, observational study examined the patient electronic health record database (Private Source 42) that was supplied by HealthVerity™ Marketplace. The dataset was converted into the OMOP Common Data Model v5. The inclusion criteria were for patients to have a dementia diagnosis in an inpatient or outpatient setting anytime between the study period; January 1, 2014 and June 30, 2019. The total data set size included 71,388 patients, but only 49,735 were included in the analysis due to exclusion because of incomplete information. A three-year period of continuous enrollment post-index and a minimum age of 18 years was required for inclusion. Patients diagnosed with heart disease, depression, or dementia during the study period were placed in the heart disease cohort, depression cohort, and the dementia cohort respectively. These patient cohort groups were further split into subgroups based on disease varieties.

### Definitions

The diseases in the study were diagnosed using the International Classification of Disease-10 (ICD-10) codes. The heart disease patient population group included patients with CAD, PAD, MI, stroke, atherosclerosis, hyperlipidemia, hypertension (HTN), and unspecified heart disease, with the respective ICD-10 codes being I25, I73.9, I21, I63, I70, E78.5, I10, and I51.9. Alzheimer's was diagnosed using the ICD-10 code G30. A diagnosis of vascular dementia was made by using code F01. The dementia group included patients with several dementia forms including AD and vascular dementia as well as dementia with Lewy bodies, and frontotemporal dementia. The following respective ICD-10 codes were used for the dementia group: G30, G31, F01, F02, and F03. This dementia group was split between patients with AD and those with vascular dementia. Patients with any form of diabetes *mellitus* were placed in their own subgroup and diagnosed using the ICD-10 codes E08, E09, E10, E11, and E13. Depression was diagnosed using the ICD-10 code F33. For examination of the patient population with depression and heart disease comorbidities, groups were formed with patients that were diagnosed with both conditions. Four separate groups were formed: patients with all-cause heart disease and depression, HF and depression, CAD and depression, and MI and depression.

### Statistical approach

The baseline demographic characteristics of the patients were assessed and compared between the patient cohort groups. Each of the individual medical conditions were analyzed individually to ascertain the impact of their presence on dementia risk. During analysis of dementia risk with a specific comorbidity, all other patient conditions were excluded to isolate the risk with that comorbidity only. The odds ratio for dementia risk was analyzed using logistic regression models that were adjusted for gender. A p value of less than 0.05 was used for statistical significance and confidence intervals were calculated at 95%. The statistical analysis was performed through the utilization of the Jamovi v2.2 platform^13^.

## RESULTS

### Participant characteristics

A total of 49,735 people were included in the final examination (age: 69.4±11.1 years; 52.7% male and 47.3% female). The ages of the patients ranged from 18 to 92 years. Out of the patient population, 13,294 (26.7%) had heart disease as their diagnosis; 2,978 (6%) had depression; 1,277 (2.6%) had a diagnosis of both heart disease and depression. Other cardiovascular disorder patient groups included 41,001 (82.4%) patients with hypertension; 33,998 (68.4%) with hyperlipidemia; 25,563 (51.4%) with heart failure; 6,341 (12.7%) with PAD; 5,256 (10.6%) with CAD; 5,205 (10.5%) with MI; and 1,718 (3%) with atherosclerosis. A total of 15,824 (31.9%) patients had some form of diabetes *mellitus*; 1,043 patients (2.1%) had dementia; 190 (0.4%) had AD; and 126 (0.3%) had vascular dementia. Study findings are summarized in Table 1.

**Table 1:** Characteristics of the study participants, n (%).

Patient characteristics		Included in analysis (n=49,735)
Age at initial visit (mean±SD; range)		69.4±11.1 years; 18–92
Sex		
	Male (%)	26,235 (52.7)
	Female (%)	23,500 (47.3)
Comorbidities		
	Hypertension (%)	41,001 (82.4)
	Hyperlipidemia (%)	33,998 (68.4)
	Heart failure (%)	25,563 (51.4)
	Diabetes *mellitus* (%)	15,864 (31.9)
	Heart disease (%)	13,296 (26.7)
	Peripheral artery disease (%)	6,341 (12.5)
	Coronary artery disease (%)	5,256 (10.6)
	Myocardial infarction (%)	5,205 (10.5)
	Depression (%)	2,978 (6)
	Atherosclerosis (%)	1,718 (3)
	Dementia (%)	1,043 (2.1)
	Alzheimer's disease (%)	190 (0.4)
	Vascular dementia (%)	126 (0.3)
Depression with a comorbidity		
	Depression with heart disease (%)	1,277 (2.6)
	Depression with heart failure (%)	1,810 (3.6)
	Depression with CAD (%)	557 (1.1)
	Depression with MI (%)	418 (0.8)

Abbreviations: n, sample size; SD, standard deviation; CAD, coronary artery disease; MI, myocardial infarction.

### Dementia risk with cardiovascular comorbidities or depression

Patients with heart disease had greater odds of dementia (odds ratio — OR 1.87, confidence interval — 95%CI 1.65–2.12, p<0.001), AD (OR 1.94, 95%CI 1.45–2.59, p<0.001), and vascular dementia (OR 1.65, 95%CI 1.15–2.37, p<0.007). Analysis of other types of heart disease yielded similarly increased odds of having dementia, AD, or vascular dementia. Depression was the variable with the highest odds for dementia. Patients who had a diagnosis of depression had considerably greater odds for having dementia (OR 3.95, 95%CI 3.37–4.62, p<0.001) and AD (OR 3.93, 95%CI 2.75–5.61, p<0.001) as well as fivefold greater odds of vascular dementia (OR 5.47, 95%CI 3.65–8.18, p<0.001). Diabetes *mellitus* patients also had a greater dementia risk (OR 1.42, 95%CI 1.25–1.61, p<0.001), AD risk (OR 1.37, 95%CI 1.02–1.84, p<0.034), and had a greater vascular dementia risk (OR 1.78, 95%CI 1.25–2.52, p<0.001). Study findings are summarized in Table 2.

**Table 2:** Logistic regression table of dementia risk with selected comorbidities.

	Comorbidities	Odds ratio	p-value	95%CI
All-cause dementia risk with selected comorbidities	Heart disease	1.87	<0.001	1.65–2.12
	Heart failure	1.41	<0.001	1.25–1.60
	MI	1.37	<0.001	1.15–1.65
	Atherosclerosis	2.06	<0.001	1.61–2.64
	CAD	1.72	<0.001	1.46–2.04
	PAD	1.82	<0.001	1.56–2.12
	Hyperlipidemia	1.39	<0.001	1.21–1.60
	Hypertension	1.50	<0.001	1.25–1.80
	Diabetes *mellitus*	1.42	<0.001	1.25–1.61
	Depression	3.95	<0.001	3.37-4.62
Alzheimer's risk with selected comorbidities	Heart disease	1.94	<0.001	1.45–2.59
	Heart failure	1.25	0.134	0.93–1.66
	MI	1.24	0.251	0.84–1.97
	Atherosclerosis	2.23	0.005	1.28–3.81
	CAD	2.07	<0.001	1.44–2.98
	PAD	1.86	<0.001	1.31–2.64
	Hyperlipidemia	1.40	0.043	1.01–1.95
	Hypertension	1.62	0.035	1.04–2.52
	Diabetes *mellitus*	1.37	0.034	1.02–1.84
	Depression	3.93	<0.001	2.75–5.61
Vascular dementia risk with selected comorbidities	Heart disease	1.65	0.007	1.15–2.37
	Heart failure	1.22	0.267	0.86–1.74
	MI	2.09	0.001	1.34–3.27
	Atherosclerosis	2.93	< 0.001	1.61–5.31
	CAD	2.18	< 0.001	1.40–3.38
	PAD	2.37	< 0.001	1.59–3.54
	Hyperlipidemia	1.58	0.03	1.04–2.40
	Hypertension	1.36	0.243	0.81–2.26
	Diabetes *mellitus*	1.78	0.001	1.25–2.52
	Depression	5.47	< 0.001	3.65–8.18

Abbreviations: CI, confidence interval; MI, myocardial infarction; CAD, coronary artery disease; PAD, peripheral artery disease.

### Dementia risk with both cardiovascular comorbidities and depression

Patients with depression and a cardiovascular comorbidity had significantly higher odds of having dementia. Patients with a history of heart disease as well as depression had a three times greater dementia risk (OR 3.47, 95%CI 2.76–4.36, p<0.001) and Alzheimer's disease risk (OR 3.23, 95%CI 1.93–5.41, p<0.001). These participants also had a greater than fivefold risk vascular dementia (OR 5.51, 95%CI 3.04–10, p<0.001). Patients who had HF and depression had fourfold higher odds of having dementia (OR 3.9, 95%CI 3.20–4.73, p<0.001), over three times higher odds of having AD (OR 3.34, 95%CI 2.08–5.36, p<0.001), as well as over five times increased odds of vascular dementia (OR 5.68, 95%CI 3.41–9.46, p<0.001). Patients with a diagnosis of CAD and depression had a threefold higher likelihood of having dementia (OR 3.56, 95%CI 2.51–5.05, p<0.001), approximately threefold higher likelihood to have AD (OR 2.89, 95%CI 1.34–6.17, p<0.007), and over five times higher odds of vascular dementia (OR 5.29, 95%CI 2.35–11.91, p<0.001). Patients who have had an MI and have depression had a greater than threefold risk of dementia (OR 3.57, 95%CI 2.37–5.37, p<0.001), approximately six times more likely to have AD (OR 5.82, 95%CI 2.47–13.74, p<0.001), and had eight times higher odds of having vascular dementia (OR 8.08, 95%CI 3.56–18.38, p<0.001). Study findings are summarized in Table 3.

**Table 3:** Logistic regression table of dementia risk in patients with both depression and a comorbidity.

	Comorbidities	Odds ratio	p-value	95%CI
All-cause dementia risk with depression and selected comorbidities	Heart disease and depression	3.47	<0.001	2.76–4.36
	Heart failure and depression	3.89	<0.001	3.20–4.73
	CAD and depression	3.56	<0.001	2.51–5.05
	MI and depression	3.57	<0.001	2.37–5.37
Alzheimer's risk with depression and selected comorbidities	Heart disease and depression	3.23	<0.001	1.93–5.41
	Heart failure and depression	3.34	<0.001	2.08–5.36
	CAD and depression	2.89	0.007	1.34–6.17
	MI and depression	5.82	<0.001	2.47–13.74
Vascular dementia with depression and selected comorbidities	Heart disease and depression	5.51	<0.001	3.04–10.0
	Heart failure and depression	5.68	<0.001	3.41–9.46
	CAD and depression	5.29	<0.001	2.35–11.91
	MI and depression	8.08	<0.001	3.56–18.38

Abbreviations: CI, confidence interval; CAD, coronary artery disease; MI, myocardial infarction.

## DISCUSSION

In this study, it was demonstrated that patients who have heart disease and depression had a statistically significant higher risk of having dementia, as well as Alzheimer's disease and vascular dementia, compared to those patients who only had heart disease. Patients with depression and who had an MI had the highest AD and vascular dementia risk. However, heart failure was the highest risk factor for all-cause dementia. To the best of our knowledge, this is possibly the first and only study that evaluated dementia risk in patients who have both depression as well as a cardiovascular disorder.

This study corroborated previous studies that found an association between AD and various comorbidities including cardiovascular diseases and diabetes *mellitus* ^14,15^. Furthermore, this study observed an amplifying effect from heart disease and comorbid depression on dementia risk in patients. Patients with heart disease had an elevated dementia risk, but that risk was a lower one compared to the group of patients with both depression and heart disease. Atherosclerosis was the form of heart disease that had the highest dementia risk in the dementia categories analyzed. In this study, atherosclerosis was the highest cardiovascular risk factor for vascular dementia. Previous studies, including Atherosclerosis Risk in Communities (ARIC), identified atherosclerosis as a risk factor for dementia^16,17^. Proposed mechanisms through which atherosclerosis can induce dementia include cerebral hypoperfusion due to vessel occlusion and altered blood flow, neurovascular unit damage, oxidative stress due to formation of reactive oxygen species from the atherosclerotic plaques, and dysregulation of miRNA expression^18^. The presence of heart failure or an MI increased AD risk, but these results were statistically insignificant. A 2006 population-based cohort study did find a higher risk of dementia and AD in elderly patients with both heart failure and low diastolic blood pressure^19^. The study also found that usage of anti-hypertensive drugs was correlated with reduced dementia risk. Similarly, heart failure and hypertension increased vascular dementia risk, but the result was not statistically significant. As with atherosclerosis, the cerebral hypoperfusion observed in heart failure and other heart conditions is an important mechanism in dementia development in those patients^20^. Diabetes *mellitus* increased dementia risk with the highest risk being vascular dementia. This corroborated previous studies that found that DM had a higher vascular dementia risk than AD^21,22^. The individual comorbidity with the highest risk factor for dementia was depression, the risk being greater than threefold. Both late-life and early-life depression have been associated with a greater risk of dementia including AD and vascular dementia^23,24^. Multiple physiological factors seen in depression have been associated with dementia. Chronic inflammation is among the most important mechanisms linking depression to impaired cognition; reduced cerebral blood flow and cerebral small vessel disease are also contributing factors^25,26^. This evidence suggests that depression may possibly be a modifiable risk factor for both preventing or delaying the onset of dementia.

The results of our study indicate that the management of these comorbidities may possibly decrease the risk of dementia or AD in patients. Recent research has found that up to 40% of all cases of dementia may be delayed or prevented through the proper management of comorbid conditions^27^. This may have significant healthcare policy implications regarding depression screening and treatment and the promotion of physical and dietary choices to mitigate heart disease risk and severity. However, a greater amount of research is needed to evaluate the efficacy of pharmacotherapies to manage heart disease and depression in reducing dementia risk. Limitations to the study include its retrospective nature and inability to determine when patients were diagnosed with their comorbidities, as well as a lack of data to evaluate if patients were currently accepting treatment for their comorbid conditions.

In conclusion, having depression as well as heart disease increases the likelihood of having dementia, including AD and vascular dementia. Both depression and heart disease are treatable, indicating a possibility of mitigating dementia risk if they are properly managed. Thus, further research is warranted to investigate the impacts of heart disease and depression management on the risk of dementia.
